# How is knowledge shared in Public involvement? A qualitative study of involvement in a health technology assessment

**DOI:** 10.1111/hex.13001

**Published:** 2019-11-29

**Authors:** Emma J. Cockcroft, Nicky Britten, Linda Long, Kristin Liabo

**Affiliations:** ^1^ NIHR Applied Research Collaboration (ARC) South West Peninsula University of Exeter Medical School Exeter UK; ^2^ Peninsula Technology Assessment Group (PenTAG) University of Exeter Medical School Exeter UK

**Keywords:** experiential knowledge, health technology assessment, hybrid knowledge, public and patient involvement

## Abstract

**Background:**

Public involvement in research is seen as a quality marker by funders. To understand the process and impact of involvement, more in‐depth studies are needed on how members of the public contribute in meetings with researchers.

**Objectives:**

This study aimed to observe and reflect on what is said by public advisers in involvement. We wanted to understand (a) what knowledge and experience is shared during research meetings, and (b) how this knowledge is shared with researchers.

**Methods:**

Data were collected in November 2016 as part of the public involvement in a health technology assessment of lung cancer screening using low‐dose computed tomography. Three meetings were audio recorded and observed with the purpose of understanding how members of the public contributed during the meetings. Audio recordings were transcribed verbatim and data analysed using a thematic approach, with the coding framework developed inductively. We also included reflections from a community drop‐in session.

**Results:**

Members of the public brought three different ‘sources’ of knowledge and experience to meetings with researchers: direct lived personal experience; learnt knowledge; and the experience and values of others. The data suggest that group settings allow for dynamic discussions and sharing of different types of knowledge.

**Conclusion:**

Group‐based involvement meetings allow for the synergistic combination of individual knowledge and experience. This gives researchers a broader understanding of the topic, which can be the vehicle for patient impact on the research. A combination of group meeting and community drop‐in can enable more varied input into research planning and conduct.

## 1 INTRODUCTION

Patient and public involvement is a broad term used to describe the involvement of patients, carers and members of the public in research decision‐making and is distinct from that of participating as a subject who provides research data. It can be defined as research being carried out ‘with’ or ‘by’ members of the public, rather than ‘to’, ‘about’ or ‘for’ them.^1^ In recent years, involvement of and collaboration with patients and members of the public is seen as a quality marker by research funders in the UK and elsewhere. Involvement is thought to lead to better quality research with more clinical relevance due to the inclusion of the unique perspective of patients and members of the public.^2^, ^3^, ^4^, ^5^, ^6^ Involvement can happen in all stages of research, and patients and members of the public can have an advisory role, collaborate with researchers or control the research.^7^, ^8^ Despite increased attention to involvement of patients and the members of the public in research, little is known about *how* involvement *works.* More theoretical underpinning or conceptualization of involvement is needed.^9^, ^10^

Patient and public involvement can be justified on a democratic or ethical basis, or on the foundation that it improves the quality of research.^11^, ^12^ It is the latter justification which this article is anchored in.^13^, ^14^ Patients and members of the public are invited to the research space to provide ‘lay’ or ‘experiential’ knowledge. This knowledge is different to, as important as and complementary to, scientific or professional knowledge.^3^, ^15^, ^16^ People's experiential knowledge can be multifaceted, gained from living with a health condition, experiencing health care or caring for someone with a condition, and complements the researchers’ academic or scientific knowledge.^17^ This ‘experiential knowledge’ can also relate to other aspects of life, such as being a parent, a smoker or unrelated professional experience. Proponents of patient and public involvement argue that this additional knowledge can create a more holistic understanding of complex health problems, thereby improving research relevance and validity.^18^, ^19^

Traditionally, experts were deemed experts due to their position, rank and education.^16^ For example, medical doctors and professors are certified experts, because they have formal qualifications which certify their knowledge. Experiential expertise of patients does not carry this ‘certification’ but is no less important to research. One problem of extending the definition of expertise in this way relates to the question of how far one should go when opening up technical decision making to experts who are experts by experience rather than certification.^18^ In the case of patient and public involvement, this particularly relates to the ‘public’ involvement. What is the naïve or lay perspective that we seek when inviting members of the public? What constitutes knowledge in patient and public involvement, and does knowledge equate to experience, and vice versa?

In the context of this paper, a member of the public was someone being a smoker or a family member of a smoker, as the HTA was about screening smokers for lung cancer. In addition, a member of the public is also someone who does not have the perspective of technical research knowledge. When involving members of the public researchers seek both knowledge gained from lived experience and lack of technical research knowledge.

A number of terms have been used to describe experiential knowledge, including ‘lay beliefs’ and ‘lay knowledge’. They all characterize this knowledge as being rooted in everyday experiences of health and illness. Stacey preferred the term ‘people knowledge’, stating that its crucial characteristics are that it is informal, experiential and mostly unwritten.^20^ Williams and Popay pointed out that lay knowledge provides an epistemological challenge to expert knowledge by challenging the impartiality of the latter, as well as a political challenge to the institutional power of medical knowledge.^21^ Research about lay knowledge has demonstrated its role in understanding the meanings of illness, and in particular the impact of illness on everyday life, and the significance of symptoms and experiences for individuals. Williams and Popay (1994) identified four themes: the diversity of lay knowledge; its internal consistency and coherence despite its differences from medical knowledge; its biographical nature; and the fact that lay knowledge is culturally framed within certain shared systems of belief. In relation to patient and public involvement, we might therefore expect diversity of responses linked to participants’ diverse experiences and biographies, and some reference to shared understandings. We might also expect some challenges to researchers’ assumptions about the nature of the problem, in this case smoking and lung cancer, and the best way to design screening programmes.

## 2 AIM

This study investigated public involvement in research; specifically, what is said during involvement meetings and the interactions between meeting participants. Our primary aims were to understand:

1. What knowledge and experience was shared at research meetings and a community drop‐in session.
2. How public advisors shared this experience with each other and with the researchers.

A secondary aim was to give a case example and description of how to involve members of the public in a health technology assessment.

## 3 INVOLVEMENT CONTEXT

The practice of patient and public involvement is shaped by its context. Below we outline the context of this particular case of involvement in research.

Data for this research were collected during three group‐based public involvement meetings (involvement format one) and one community drop‐in session (involvement format two) to inform a health technology assessment (HTA) of screening for lung cancer using low‐dose computed tomography. Public involvement for this project was initiated at the request of funders and aimed to help inform the systematic review protocol for the HTA, and to aid the HTA researchers’ understanding of (a) important outcomes, particularly those relating to quality‐of‐life and (b) the broader social acceptability of such a screening programme. The involvement activity reported in this paper took place at the start of the research, to inform the review protocol and researchers’ understanding of outcomes. There was additional involvement of one person in a larger stakeholder meeting later in the process which is not reported here.

The involvement plan was devised by four researchers. Two from the HTA team (LL and another researcher from this group) and two from a research team that regularly plans and facilitates involvement (EC and KL). After discussions, we decided to invite people who would be recipients of this screening programme if implemented, in particular current and past smokers aged 50 and over. The decision to involve current and ex‐smokers rather than people who had a lung cancer diagnosis was taken, as they would be the target group of the potential screening programme.

The opportunity to inform this HTA on lung cancer screening was advertised to current and ex‐smokers through research involvement groups at the university, newspaper adverts, posters at doctors’ surgeries, and at a community centre situated locally in an area of deprivation (see Appendix S1). Two different involvement formats were used.

### 3.1 Involvement format one (structured group meetings)

The format described below was used for three separate group meetings, each with the same structure. Two of these meetings were held at the university and one at a local community centre. All participants were reimbursed travel expenses and received a £25 thank you payment for their time.

No prior knowledge or understanding of research was required before the meeting. Each meeting lasted ~2 hours and began with giving each person, including the facilitators, the opportunity to introduce themselves and their background. This allowed the group to get to know each other and gave everyone a chance to speak early on. The meeting included an introductory session to explain health technology assessment, and two structured discussions on outcomes and possible barriers to attending screening. The introductory section of each meeting was facilitated by LL and involved a video explanation of ‘what is a health technology assessment?’ (https://www.youtube.com/watch?v=mwQVIRQ2C4U) followed by an opportunity to ask questions of members of the lung cancer screening HTA team.

**Figure 1:**
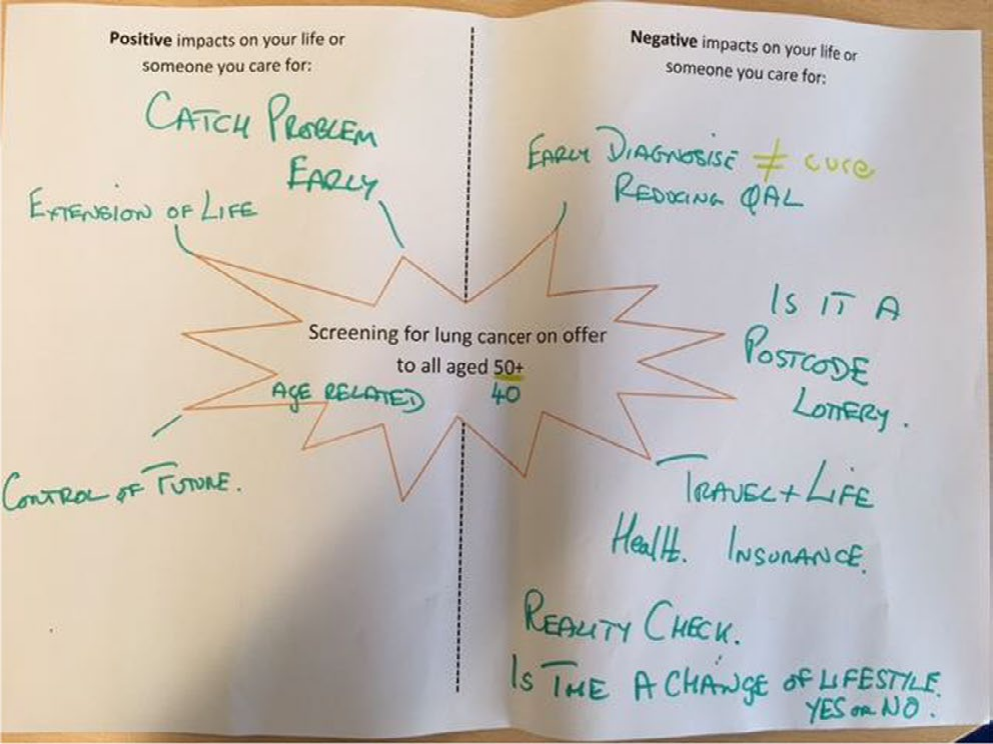
Example of completed outcome star after small group discussion

**Figure 2:**
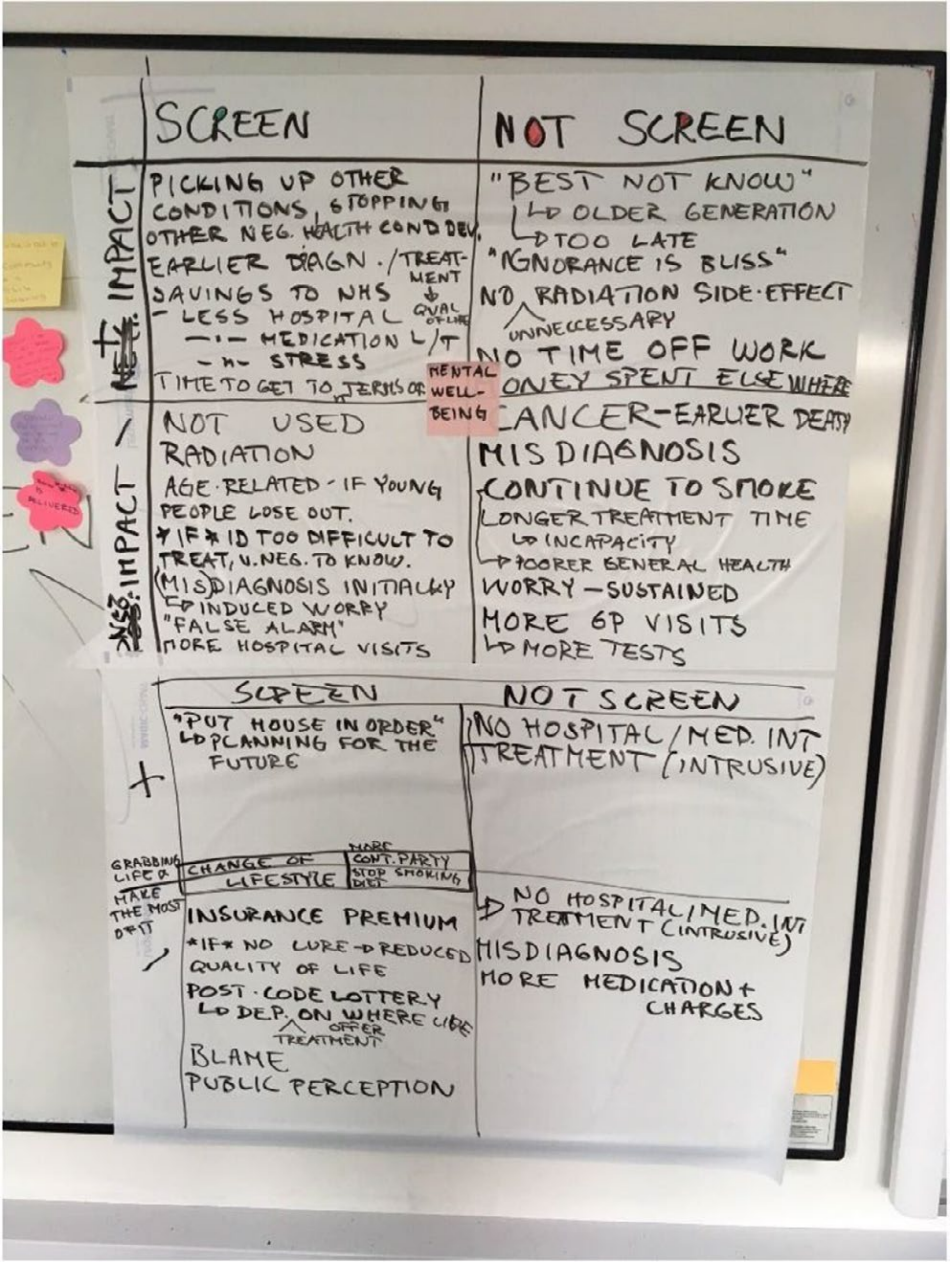
Example white board where points raised in whole group discussion were summarized

Next followed a structured discussion facilitated by KL to hear people's views on screening outcomes. Members of the public were encouraged to think about the impact that screening for lung cancer might have on their lives (both positive and negative). This was initially in pairs, using ‘outcome stars’ (see Figure 1, for example) with hypothetical situations of screening offered or not offered to current or ex‐smokers over 50 years old, and followed by a whole group discussion where ideas were recorded on a whiteboard (Figure 2). The combination of small and large group discussions was intended to encourage members of the public to contribute even if they were uncomfortable in larger groups, while also sharing views across the whole meeting.

**Figure 3:**
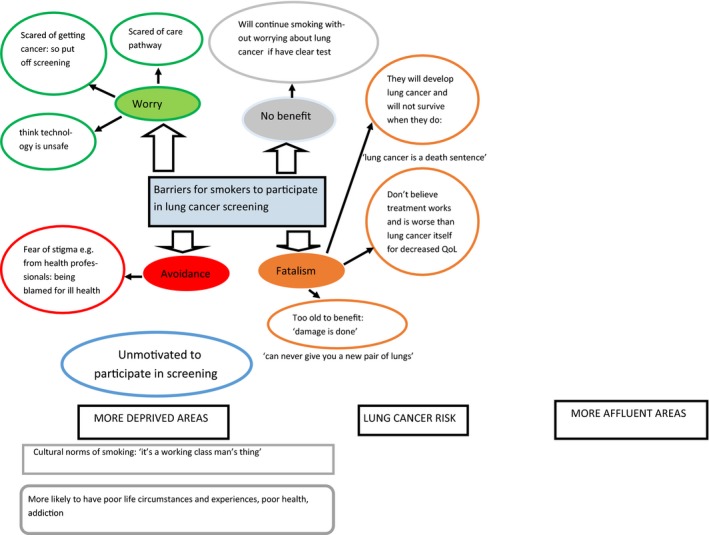
Example of visual model based on relevant qualitative research findings to stimulate an open whole group discussion

Finally, members of the public were encouraged to discuss the perspective of smokers/ former smokers from deprived areas on the introduction of a lung cancer screening programme. Discussion was aided by using data from a qualitative interview study published in 2017.^22^ LL facilitated discussions by presenting the findings of this study as visual models (Figure 3).^23^ LL used these visual models during the third part of the workshops to stimulate an open group discussion on barriers, why people may or may not attend screening, and the social acceptability of the programme.

The groups were run strictly to time, and the facilitators (KL and LL) aimed to moderate the groups so that everyone had the opportunity to speak. At the beginning and throughout the groups, KL and LL emphasized the non‐judgemental nature of the discussion, and sometimes shared their own experiences related to the conversations. Throughout the meetings both KL and LL took notes, first ensuring that they had understood points correctly by double checking with public advisors. The meetings included refreshments and where followed by lunch to allow discussion to continue in a more informal setting.

### 3.2 Involvement format two (community drop in)

**Figure 4:**
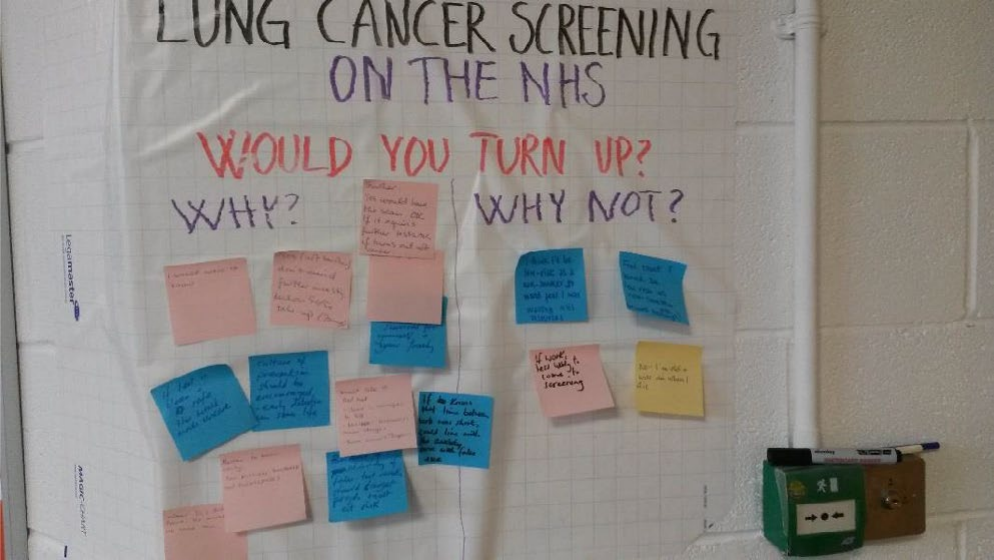
Example whiteboard question during community drop‐in session

The second format of involvement took place at a community centre during a regular coffee morning, at a time when people collected food from a food bank located within the centre. The community centre is in a deprived residential area of a small city. Three of the researchers (LL from the HTA research team and KL and EC from the Involvement team) discussed lung cancer screening using low‐dose CT one‐to‐one with members of the public coming to the coffee morning. Members of the public were invited to write their thoughts on post‐its and stick them to a board. This allowed members of the public using the community centre to share their thoughts on outcomes, quality of life and social acceptability of lung cancer screening in a format that could later be shared with the wider HTA research team (Figure 4). The discussions were intentionally informal, and the researchers were mindful of inviting comments and talk, but not pushing the topic on to anyone who preferred not to engage.

## 4 RESEARCH METHODS

### 4.1 Data collection

Ethical approval for the study was granted by the University of Exeter Medical School Research Ethics Committee (Nov16/D/100∆1). Written consent was obtained from participants before the start of each meeting, after reading an information sheet about the patient and public involvement study as well as discussion with EC. Data were collected during November 2016.

Data included audio recordings of group meetings, group meeting observation notes, and facilitator/participant reflections before and after each meeting. In total data consisted of 5 hours 50 minutes of recordings, 2 observations, 12 researcher reflections (3 from each meeting) and 8 participant reflections (4, 3 and 1 from groups 1, 2 and 3, respectively). Reflections were captured by filling in report cards adapted from Popay et al 2014^24^ to assess views about the meetings (see Appendix S2).

### 4.2 Participants

*Group meeting 1*: Nine people: members of an established involvement group. Ex‐smokers and smokers, and some who had lost a family member to a smoking‐related illness.

*Group meeting 2*: Five people: four members of the public who had responded to a call for involvement in this study in local newspapers or adverts in GP surgeries, and one member of an established involvement group. Ex‐smokers and smokers, one of whom had also lost a close family member to a smoking‐related illness.

*Group meeting 3:* Four people: three members of the public recruited through links with a local community centre and one member of an established involvement group. Ex‐smokers and people who had lost family members to a smoking‐related illness.

Collectively, we involved a total of 17 members of the public (11 female) all aged over 18 and mainly aged >40.

*Format two, Community drop‐in session:* The number of members of the public spoken to or who posted their views on the board were not formally recorded but included smokers, ex‐smokers and never smokers.

*Researchers:* All researchers were non‐smokers, but had experience of family members smoking and having smoking‐related illness.

### 4.3 Data analysis

All group meetings were audio recorded and transcribed verbatim. Transcripts were anonymized using descriptive codes. EC transcribed audio recordings of meetings, which were checked by LL. All data were imported to NVivo and analysed using this software (version 12 QSR International). Our coding framework started with our research questions and then further developed inductively as described by Miles and Huberman.^25^

Firstly, EC and KL familiarized themselves with meeting transcripts, observation notes and researcher/participant reflections with a focus on aspects relating to lived experience, group dynamics and facilitation of meetings. Any pertinent thoughts during this phase of analysis were recorded as annotations (analytical notes) in NVivo and referred to in subsequent meetings. EC and KL then met to compare analytical notes from their individual analysis to refine the coding framework. Next, EC and KL re‐read and coded the transcripts, report cards and observation notes. EC and KL then compared their coding, discussed further emerging codes and refined existing codes. Following this, EC and KL re‐read data and updated their analysis. Third, EC and KL discussed the codes and how they grouped into themes.^25^ These themes were independently verified by LL and discussed in a further meeting. Themes were compared across the different meeting formats. Quotations are presented verbatim in italic text. They were chosen to illustrate the themes, with each quotation identified with participant gender, smoking status and meeting number.

## 5 FINDINGS

The themes identified are discussed in more detail below and consist of: source of knowledge, how knowledge is shared, and group dynamics.

### 5.1 Source of ‘experiential knowledge’

The data describe three different ‘sources’ of knowledge that arose during the group discussions: direct lived personal experience; learnt knowledge; and the experience and values of others.

In patient and public involvement meetings, members of the public are usually invited due to their personal lived experiences of the research topic. This sharing of personal experience was noted in all three of the workshops we ran for this project. Members of the public shared very personal stories without undue prompting and seemed willing to do so, also in front of strangers. The meeting design and facilitation style, which included facilitators sharing their own personal experiences, was carried out with this aim in mind, with data suggesting that this was successful.

“about two years ago I was walking down the road, and I coughed and went like that and it was all blood, I thought oh my god, just I don’t know what to do, and I just phoned the doctors, I said, “Can I make an appointment?” “I’m sorry, no appointments today [own name],” I thought, oh my god, what should I do because it really alarmed me, so I thought, oh my god, so I took myself up to the walk‐in centre in [name of road], and she said, “Oh I think we better get you a taxi to go up the hospital.” Female 8, ex‐smoker, 2nd meeting

In the example above, the person is relating a very personal and particular encounter relevant to the topic of lung cancer screening. Her account went into some detail, including a distressing comment by a medical consultant.

In addition to this ‘expected’ contribution of direct personal experiences members of the public shared different *types* of experiential knowledge which were related to broader aspects of life and might be ‘learnt’ rather than directly experienced.

“For me growing up I felt a cultural change when Roy Castle got cancer and I think society changed” Female 9, ex‐smoker, 2nd meeting

“They are saying more and more that in western society diabetes two is indicative of people, because of what they eat.” Female 3, smoker, 1st meeting

The first quote, referencing the celebrity Roy Castle, was not the only one relating to someone famous who was affected by lung cancer, and the subsequent knowledge gained from publicity surrounding such cases. The second quote shows awareness of individual choice on health status, and how news items influence people's knowledge on a more general topic. These are examples of how members of the public brought knowledge from external sources to the discussion on lung cancer screening HTA. We had invited members of the public to the meetings based on their smoking behaviour or experiences of losing family members to smoking‐related illnesses, but they brought knowledge and experience that went beyond their own personal circumstances, as well as beyond the topic of lung cancer.

Members of the public also brought with them the views and experience of others. This was most often in the form of sharing the values of family and friends and the experiences that comes with those values.

“my father when he was diagnosed. Umm he wasn’t with my mother any longer…. so he went out and got himself married again…. Got himself a new life …. Umm so for him it was a very positive thing because he knew how long he’d got and he was very conscious of making every moment count. I would say the negative impact was that actually when we went to his funeral which was [elsewhere], they didn’t mention any of his previous life.” Female 3, smoker, 1st meeting

“… people that I’ve spoken to about breast screening don’t want to go because they don’t want to find out, and that was their answer” Male 6, ex‐smoker, 3rd meeting

In group meeting discussions, members of the public tended to support of each other's views and generally to support lung cancer screening being introduced as a preventative measure. However, during the community drop‐in sessions, when researchers spoke to members of the public one‐to‐one, conversations tended to be more open and upfront, as highlighted in the post‐session reflections of the researchers.

**Researcher 1: **“Talking one‐to‐one in a community centre environment was more relaxed and personal than previous group discussion formats, and allowed more intuitive prompting about aspects of quality of life relating to the technology eg concerns about re‐testing and safety. This approach elicited views previously un‐voiced.”

### 5.2 How content of knowledge was presented in meetings

The ‘knowledge’ shared during meetings was expressed in a variety of different ways from subtle hinting to being forthright and, occasionally, judgemental. Members of the public also shared knowledge based on reasoning derived from reflection on past experience relating to the topic. We found that on occasions participants would share a possibly controversial opinion in a subtle rather than upfront manner, *hinting* at a point and not necessarily taking ownership of it.

“so, redirecting the NHS funds to somewhere where it could be more used. Now that’s not my opinion I’m just saying that” Female 4, smoker, 1st meeting

This is important for facilitators and researchers as it shows that important comments are not always obvious and may be brushed away if only *hinted* at. Conversely, in contrast to *hinting* we observed really *forthright* comments, said with confidence.

“Well if it’s too expensive it’s too expensive, it’s like drugs isn’t it, highly desirable drugs that cost £2,000 a pop, and they can’t do it, just can’t do everything. So it’s tough. And we are going to all die, and if we don’t die of lung cancer or some other…” Female 11, ex‐smoker, 3rd meeting

Some of the comments during meetings could have also been described as *judgemental*. The example below was in response to discussion about people not turning up to screening appointments.

“Yeah, just inadequate…they’re dim’…’ Make every excuse why they don’t… it’s the same people who miss buses and don’t turn up for, can’t rely on things, it’s always somebody else’s fault”. Female 11, ex‐smoker, 3rd meeting

This direct way of sharing their views was also observed in the community drop‐in session, with reflections from one researcher stating:

**Researcher 3** ‘Despite the less formal facilitation I felt it enables us to have more in‐depth conversations and the members of the public were happy to give their honest views without fear of judgement from others’.

Finally, it was apparent that participants shared the content of knowledge in a *reasoned* way, thinking through the implications of past experience to build an argument and help make a point relevant to the discussion.

‘No, my wife wouldn’t, she won’t even go in and get tested, she had a breast x‐ray about ten years ago, they keep writing to her asking her to come back in, she won’t, and I’ve said to her, ‘Have you got any lumps there?’ ‘Well that’s why I’m not going in because there isn’t any,’ ‘Oh alright, suit yourself, do it your way,’ but the thing is, you know, if she did suddenly develop breast cancer then the whole family is going to suffer, which is you know, it’s offered, she’s not taking it, so that’s a negative on the offered one. It’s offered and people not taking it up, that’s real bad seeing that.’ Male 6, ex‐smoker, 3rd meeting

### 5.3 Group dynamics

In the workshop‐based meetings described here, group dynamics will play a part in whether members of the public feel able to express their views and experiences, and whether the HTA researchers feel the discussion is relevant to their work. The meetings that were part of this research were designed and facilitated with a set intention. Members of the public first worked in small groups on specific questions before they joined a discussion with the whole group. Participants were observed agreeing and supporting others’ views when sharing personal experiences and knowledge.

Researcher 1: So if you take up the offer, going for lung cancer screening, how will that affect your quality of life?

Female 11, ex‐smoker: Well because if it's effective early treatment it's going to mean I’m not going to be in pain and agony and [gasping].

Researcher 1: Yeah, early detection.

Researcher 2: But if you have it, even if it's early detection you do then have to go through the treatments, so that might still impact.

Female 11, ex‐smoker: Yes, but it would be less of an impact than waiting until it was too late.

Researcher 2: Yeah.

Male 5, non‐smoker: Yeah.

Researcher 2: So it's a weighing up of that.

Male 5, non‐smoker: Early detection, number one early detection.

Such supportive conversations amongst members of the public, and between researchers and members of the public, appeared to further facilitate the sharing of personal stories in the group. This was further confirmed in the after meeting reflections where members of the public stated how they were able to voice their own views on the topic.

“I felt the environment was conducive to the topic. It felt “safe” to air my views and the “ground rules” really helped set the scene.” Female, smoker, 1st meeting

“I felt totally able to give my views and enjoyed hearing other people's views” Female, non‐smoker, 1st meeting

“I was able to put my points of view across whenever asked or needed to.” Male, ex‐smoker, 2nd meeting

## 6 DISCUSSION

Through audio‐recording and observations of meetings with members of the public, we have analysed the kinds of experiences and knowledge members of the public brought to the research, in their capacity as public advisors. The purpose of our analysis was to understand better what members of the public bring to discussions about research so that in turn we can improve and enhance these collaborations. Our study highlights (a) the varying sources of experiential knowledge shared at meetings; (b) the subtle differences in how knowledge is shared; (c) how group dynamics can play a part in whether members of the public feel able to express their views and experiences, and how a meeting is run; and (d) how people can be involved in research in different formats and levels of input.

### 6.1 Involvement methods

From a practical perspective, this paper illustrates two examples of how public involvement can be conducted to inform research. Specifically, we describe a way of facilitating structured group discussions on outcomes in research by discussing impacts on day‐to‐day life in different scenarios (see Figures 1 and 2). In addition, we provide an example of a ‘drop in’ involvement session, which allows researchers to speak briefly to a variety of different people who might not want to attend a bespoke workshop, but welcome the opportunity to share views to influence research. As such, we add to a growing body of work that illustrates innovative ways of facilitating discussions on research and gaining input from members of the public.^26^, ^27^, ^28^

The one‐to‐one approach at the community centre drop‐in may have value beyond group discussion, allowing members of the public space to share more contested views and should be considered a useful addition to structured group‐based involvement meetings. The drop‐in style involvement described here might also serve as an introduction to members of the public about what involvement is, and enable them to make an informed decision on whether they would like to be more involved in research. One of the members of the public at the drop‐in later attended a group workshop and volunteered for future involvement in research based on these experiences. An additional value of the drop‐in style session is that it allows for a more democratic oversight, allowing a wider and richer range of viewpoints to be added to the research process.

An important aspect of this is the facilitation and planning of the sessions. The team who were involved were a mix of researchers from the HTA team, and specialist patient and public involvement researchers who regularly support members of the public, patients and carers to be involved in research.

### 6.2 Research findings

Our data highlight that individuals bring different types of knowledge to research and that their input is not only based on their personal and direct experience. Members of the public's knowledge include that which is learnt, opinions (which may or may not be based on experience) and the views, values and experiences of others. This corresponds to Mikhail Bakhtin's theory of ‘polyphony of voices’.^29^, ^30^ Bakhtin considers the *self* to consist of a number of autonomous ‘I’ positions, which we are able to switch between in both internal and external dialogue. In other words, the self consists of a multitude of different voices and forms of knowledge which might include ‘I’ as my mother's daughter, ‘I’ as a member of the community and ‘I’ as a student. This has also been previously reported by Popayand Williams,^15^ who suggest that members of the public often have detailed knowledge of other people as well as themselves, and an intimate knowledge about the circumstances in which they live.

In patient and public involvement, we often invite people to meetings based on relevant experience. In this project, for example, we invited members of the public who were present or past smokers, and some who had lost close family members to smoking. What we see is that members of the public bring more than this: they may wear multiple hats and bring different types of knowledge, such as something they have read, the opinion of a relative, or the experience of a relative as told and interpreted by themselves. The perspective they bring is not just simply that of a smoker but also of a smoker's ‘child’, a daughter‐in‐law, or a spouse. This suggests that the knowledge brought to research by patients and members of the public goes well beyond ‘lived experiences’ and ‘naïve’ perspectives. It further suggests that it is important to be aware of the different roles people hold and to consider how members of the public are prepared for meetings with researchers. It is likely that the lived experience (in this instance smoking status) might also influence the content of health concerns shared in meetings.

Although we advocate for a variety of approaches to involvement, including one‐to‐one discussions, our findings suggest that group meetings between researchers and members of the public are important. This is particularly significant as meetings can enable discussion of a variety of perspectives and types of knowledge. This is similar to that of focus group methodology which has been described as offering a platform for differing paradigms or worldviews.^31^, ^32^, ^33^ This may also have a negative impact by creating a herd effect where people's views fall in line with the most dominant of safe views.

We propose that discussions in group meetings can allow for an amalgamation of different types of knowledge from different people, which might complement or adjust previous knowledge. Both researchers and members of the public might leave meetings with a refreshed understanding. This new understanding is a combination of existing knowledge, which is complemented and altered by the knowledge shared by others in the meeting. It is held by the individual and may be different for each individual depending on their prior knowledge and experience. This ‘new’ knowledge is different from the sum of separately collected individual knowledge and is created as part of the context of the meeting, reflecting the discussion in the group. We believe that this might be influenced by the dynamics of the group and mediated through facilitated discussion, with the dialogue and inclusive nature of meetings an essential component that allows for rich discussion. A similar concept of hybrid knowledge has been described by Renedo et al,^34^ who detail how dialogue between researchers and members of the public can bring about hybridisation, where there is an amalgamation of knowledges which are typically found separately (eg lay knowledge and technical research knowledge), resulting in new combined understanding.

Our findings also relate to the Bakhtinian notion of dialogism and dialogical learning,^35^ where hybrid forms of knowledge are produced though dialogue and by reworking the knowledge of others. This involves actively re‐negotiating prior understanding by combining the knowledge of others with prior knowledge and values,^36^ for example knowing that stigma is an important consideration in screening programmes, but not fully considering it until its importance has been re‐emphasized by the knowledge and experience of others. For this to happen in public involvement, the researcher at the public involvement meeting needs to listen and discuss the research topic with members of the public, and in turn rework their own current understanding of this topic. This reworking through listening and discussions may be one of the routes for impact and influence of public involvement on research,^37^ which is often hard to evaluate if not captured in meeting notes and if researchers are not aware of the shift in their thinking. In the case of this HTA, this shift would be changed to the researchers’ understanding of important outcomes and social acceptability of lung cancer screening.

Collectively, these findings highlight the importance of group‐based involvement meetings, where the collective voice is more than the sum of its parts, or as described by Aristotle ‘A feast to which many contribute is better than a dinner provided out of a single purse’ 38; people provide different pieces of knowledge which generate rounded discussions and may re‐accentuate or emphasize prior understanding. Central to this suggestion of group understanding is the importance of facilitation and the influence of group dynamics and how this can influence the ways in which individuals share their knowledge and experience, allowing back and forth between people and space to listen and reflect. Planned and structured meetings allow for targeted discussions and activities designed to help sharing and mobilization of experiential knowledge within the group, and provide a safe space to challenge and discuss aspects of one's owns knowledge with others.

### 6.3 Limitations

This study adds important empirical data to our understanding of patient and public involvement. However, we acknowledge that there are limitations to this work. Firstly, because this is one particular example of involvement which focuses on the early stage of research, we cannot explore knowledge contributions from members of the public over time. Second, although we present data collected in the meetings, we cannot fully assess the impact from involvement without interviewing the researchers involved. This would be an interesting avenue for future research, testing our theory of reworked understanding after meetings and the impact this has on research.

## 7 CONCLUSION

Findings from this study show the details of experiential knowledge conveyed during public involvement meetings and give an insight into how involvement ‘works’, by highlighting what we term hybrid understanding. From a practical perspective, we show the benefits of both group meetings and one‐to‐one discussions. We propose that group meetings allow for the synergistic combination of individual knowledge and experience, which has benefit above and beyond speaking to the same people on an individual basis. We also highlight the additional benefit and ease of ‘drop in’ one‐to‐one sessions, which can allow people to share more contested views, which can add value, especially for projects which may include diverging views. We suggest the meeting facilitation is key in the running of involvement meetings, enabling and allowing for people to share openly and discuss with others. Finally, we suggest that impact from involvement is improved when researchers have direct conversations with those with relevant lived experience.

## CONFLICT OF INTEREST

None declared.

## Data Availability

The data that support the findings of this study are available on request from the corresponding author. The data are not publicly available due to privacy or ethical restrictions.
