# Supplementary material for: How is knowledge shared in Public involvement? A qualitative study of involvement in a health technology assessment
Source: Health Expect. 2019 Nov 29;23(2):348–57. doi: 10.1111/hex.13001 (PMC7104644; doi:10.1111/hex.13001)
Supplement: Supplementary file 1 [file HEX-23-348-s001.pdf]

Appendix 1: Example posters and newspaper adverts to advertise this involvment opportunity.

NIHR CLAHRC
South West Peninsula

NHS
National Institute for Health Research

UNIVERSITY OF EXETER | MEDICAL SCHOOL

Help needed to influence research on lung cancer screening

What?: We would like to hear your views on lung cancer screening to help us prioritise what is important to you. Drop by and share your views with us.

Why?: Public involvement in research ensures that the public have their say in how the research is conducted.

Where?: Community Centre, meeting room 1.

When?: **Friday 18th November** From **9:30 to 12:00.**

If you would like more information please get in contact:
Emma Cockcroft: **E.J.Cockcroft@Exeter.ac.uk**

a) Poster for community drop in session

UNIVERSITY OF EXETER | MEDICAL SCHOOL

Help needed to influence research on lung cancer screening

We are looking for **current or ex-smokers** that would like to help shape new research on lung cancer screening

---

Who?: People who currently smoke or who have quit in the last 15 years.

What?: We would like to hear your views on lung cancer screening to help us prioritise what is important from your point of view.

Why?: Public involvement in research ensures that the people who it effects have their say in how the research is conducted.

Where?: The University of Exeter Medical School, St Luke's Campus, on Heavitree Road (opposite Waitrose)

When?: **Wednesday 16th November** From **11:00 to 13:00**

You will be paid £25 for you time and reimbursed any travel costs

If you would like more information or want to register for this event please get in contact: o

Emma Cockcroft: **E.J.Cockcroft@Exeter.ac.uk** or **01392 72 2764**

Lung cancer screening research involvement
Contract Emma Cockcroft:
E.J.Cockcroft@Exeter.ac.uk
Or call: 01392 72 2764

Lung cancer screening research involvement
Contract Emma Cockcroft:
E.J.Cockcroft@Exeter.ac.uk
Or call: 01392 72 2764

Lung cancer screening research involvement
Contract Emma Cockcroft:
E.J.Cockcroft@Exeter.ac.uk
Or call: 01392 72 2764

Lung cancer screening research involvement
Contract Emma Cockcroft:
E.J.Cockcroft@Exeter.ac.uk
Or call: 01392 72 2764

Lung cancer screening research involvement
Contract Emma Cockcroft:
E.J.Cockcroft@Exeter.ac.uk
Or call: 01392 72 2764

Lung cancer screening research involvement
Contract Emma Cockcroft:
E.J.Cockcroft@Exeter.ac.uk
Or call: 01392 72 2764

b) Example poster placed in doctors' surgery

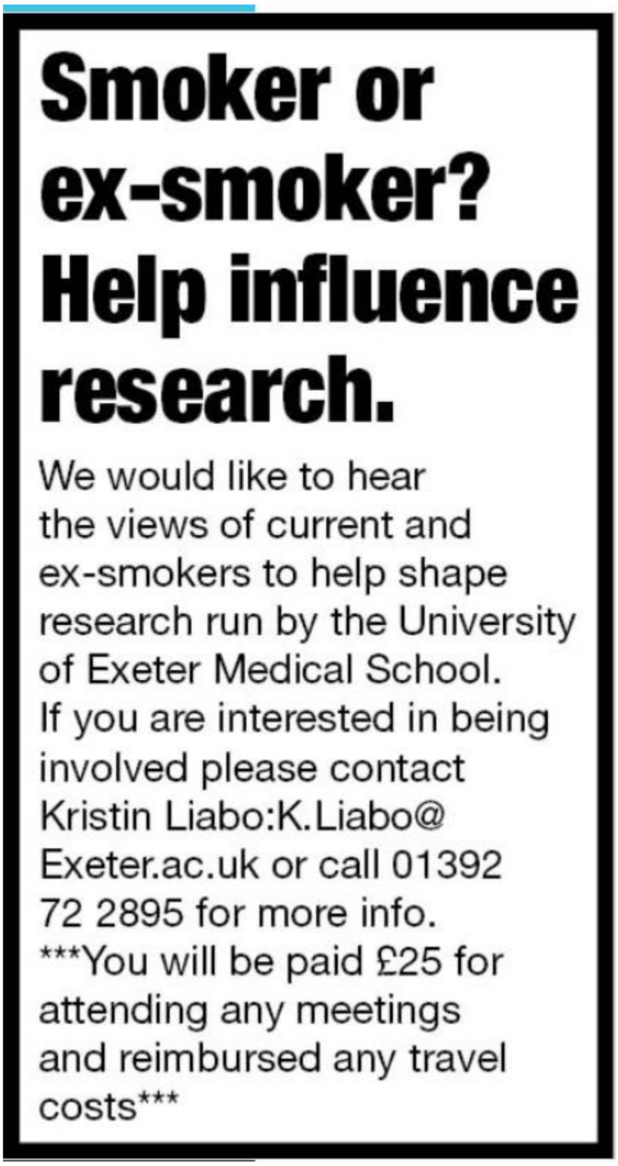

Smoker or ex-smoker? Help influence research.

We would like to hear the views of current and ex-smokers to help shape research run by the University of Exeter Medical School.
If you are interested in being involved please contact Kristin Liabo:K.Liabo@Exeter.ac.uk or call 01392 72 2895 for more info.
You will be paid £25 for attending any meetings and reimbursed any travel costs

c) Example advert placed in local newspaper
