# Supplementary material for: How is knowledge shared in Public involvement? A qualitative study of involvement in a health technology assessment
Source: Health Expect. 2019 Nov 29;23(2):348–57. doi: 10.1111/hex.13001 (PMC7104644; doi:10.1111/hex.13001)
Supplement: Supplementary file 2 [file HEX-23-348-s002.docx]

**Appendix 2: Report cards**

Contact summary form: researchers

1. What were the main issues or themes that struck you as coming from public contributors in this meeting? (Describe key concerns or points raised, identify whether this is a point raised by one person and conceived as important by you, or a point which had support across the whole group)

2.       Summarise your views about this meeting in regards to

1. The usefulness of the format/facilitation
2. How people brought their experiences to the table when discussing lung cancer screening
3. The relevance of what people said to the HTA research, and whether this has implications for the review protocol

3.       Anything else that struck you as salient, interesting, illuminating or important in this meeting? (Consider anything here that you found interesting, beyond lung cancer screening, HTA or PPI)

Contact summary form: Participants

1. What were the main things said in the discussions at this meeting? (There is no right or wrong answer – tell us what you thought were the important points coming through in the discussions)

1. Summaries your views about this meeting in regards to

- 1. What do you think about the researchers’ explanation about the research and how this was presented?
  2. How successful do you think the researchers were in making the meeting interesting? (Be honest!)
  3. Did you feel able to voice your own views when discussing lung cancer screening?

1. Anything else that struck you as interesting or important in this meeting? (Consider anything here that you found interesting, it doesn’t have to be in regards to lung cancer screening or HTA)
